# Administration of pigment epithelium-derived factor delivered by adeno-associated virus inhibits blood–retinal barrier breakdown in diabetic rats

**Published:** 2010-11-15

**Authors:** Hai Yu, Lei Chen, Jing Jiang

**Affiliations:** 1 Department of Ophthalmology, First affiliated Hospital of China Medical University, Shenyang, Liaoning, China; 2 Department of Ophthalmology, General Hospital of Shenyang Military Command, Shenyang, Liaoning, China

## Abstract

**Purpose:**

To evaluate the effect of the recombinant adeno-associated virus (rAAV) vector that expresses human pigment epithelium-derived factor (hPEDF) on reducing blood–retinal barrier (BRB) breakdown in the experimental diabetic rat model.

**Methods:**

Diabetes was induced by an intraperitoneal (i.p.) injection of streptozotocin (STZ) into 10-week-old male Wister rats. rAAV_2_-cytomegalovirus (CMV)-hPEDF was delivered into the right eyes by intravitreal injection on the first day after diabetes induction. The contralateral eyes received intravitreal injection of rAAV_2_-CMV-green fluorescent protein as the paired control. Gene delivery and expression of vascular endothelial growth factor (*VEGF*), occludin, and intercellular adhesion molecule-1 (*ICAM-1*) were determined with reverse transciptase PCR or western blotting. BRB breakdown changes were quantified by measuring albumin leakage from retinal blood vessels after an intravenous (i.v.) injection of Evans blue albumin.

**Results:**

Retinal transfection with the *hPEDF* gene construct led to sustained *hPEDF* gene expression for 6 months, significantly suppressing *VEGF* mRNA expression in the retina after 1, 3, and 6 months of diabetes induced by STZ compared with paired controls. Moreover, hPEDF dramatically reduced the levels of retinal ICAM-1 but increased the expression of occludin. Furthermore, BRB breakdown was much lower in hPEDF-injected diabetic animals in comparison with controls after 6 months.

**Conclusions:**

A single intravitreal injection of rAAV_2_-CMV-hPEDF can relieve BRB breakdown in STZ-induced diabetic rats for 6 months. The effect is associated with downregulation of retinal *VEGF* mRNA and *ICAM-1* expression and a reduction in the loss of retinal occludin induced by diabetes. The approach of gene transfer may reduce diabetic macular edema, providing long-term protection for diabetic patients at risk of macular edema.

## Introduction

Diabetic macular edema (DME) can lead to considerable vision loss in diabetic patients, especially in those with nonproliferative diabetic retinopathy (NPDR). An increase in vasopermeability, caused by the breakdown of the blood–retinal barrier (BRB), is a key factor of DME [1,2]. Although laser therapy has shown some effect on preventing partial visual loss, the current treatments for DME are far from satisfactory.

Vascular endothelial growth factor (VEGF), a hypoxia-induced angiogenic factor, is a potent vascular permeability factor that can cause hyperpermeability by inducing phosphorylation of tight junction proteins, such as occludin and zonula occludin-1 (ZO-1) [3,4]. Studies have shown that a high level of VEGF, found in the retina and vitreous of DME patients, coincided with BRB breakdown [5,6]; this suggests that VEGF may play an important role in DME formation. Recent studies have reported that pigment epithelium-derived factor (PEDF), a potent angiogenic inhibitor, was found in decreased amounts in the vitreous of diabetic retinopathy (DR) patients [7–10]. Vitreal injections of PEDF can suppress BRB breakdown and vascular permeability induced by VEGF [11], which implies that PEDF can take part in regulating vascular permeability. Based on the above, we hypothesize that an increase of retinal PEDF would antagonize VEGF and inhibit BRB breakdown in patients with diabetes.

Intravitreal injection is the best way to deliver PEDF. However, due to the short half-life of PEDF [12], multiple injections are required for treatment. However, repeated intraocular injections may cause increased risk of side effects, such as vitreous hemorrhage and retinal detachment, and may not be feasible depending upon the frequency of the injections required. Therefore, any approach that incorporates sustained drug delivery will improve efficacy and safety. Gene transfer offers an alternative means for this purpose. Recombinant adeno-associated virus vectors (AAV) have become potent gene delivery tools in a variety of animal models that mimic human retinal diseases because of a lack of pathogenicity, minimal immunogenicity, stable and efficient transfection, and the maintenance of high levels of transgene expression. Prolonged transgene expression inhibition of choroidal neovascularization has been demonstrated after intraocular injection of AAV-mediated PEDF [13]. Herein, we used AAV vectors containing genes coding human PEDF to treat diabetic rats and to test the long-term effects of intravitreal injection of hPEDF on retinal permeability.

## Methods

### Animals

Ten-week-old male Wister rats, weighing approximately 180–200 g, were purchased from the China Medical University Animal Resource Laboratory (Shenyang,China). Care, use, and treatment of all animals approved by the laboratory animal center and in this study were in strict agreement with the guidelines on the care and use of laboratory animals set forth by the laboratory animal center of China Medical University.

### Rats model of streptozotocin-induced diabetes

Diabetes was induced by an intraperitoneal injection of streptozotocin (STZ; 60 mg kg^−1^ in 10 mmol l^−1^ of citrate buffer, pH 4.4; Sigma, Shanghai, China) into Wister rats that had been fasted for 12 h. Blood from tail vein was used for blood glucose level test 48 h after injection and blood glucose levels were monitored on a monthly basis. Only animals with blood glucose concentrations higher than 16.7 mmol/l were considered diabetic. The diabetic rats without complications from intravitreal injection (such as retinal detachment, hemorrhages, infection) were divided into three groups:1 month (DM1), 3 months (DM3), and 6 months (DM6). Due to unexpected deaths, the final sample size of each group was 21 (4/25), 21 (4/25), and 20 (5/25), respectively.

### Intravitreal injection of vector

Wister rats were intravitreally injected on the first day of diabetes (the animals had a blood glucose level higher than 16.7 mM). To avoid unnecessary effects on the retina induced by some nonexperimental factors, such as vectors themselves and the injection procedure, both eyes of rats were injected with vector. The rats were anesthetized with ketamine (Shandong fangming pharmaceutical Co, Ltd, Dongming, China; 80 mg kg^−1^) by intraperitoneal injection, and the pupils dilated with tropicamide; then a 30-gauge needle on a Hamilton syringe was inserted into the vitreous cavity through the sclera just behind the limbus of the right eye, under microscopic control. Two microliters of rAAV_2_-cytomegalovirus (CMV)-hPEDF (1×10^11^ vector genomes ml^−1^; AGTC Gene Technology Company Ltd, Beijing, China) was injected into the vitreous of the right eye. The contralateral eye was injected with 2 μl of rAAV_2_-CMV-green fluorescent protein (GFP; 1×10^11^ vector genomes ml^−1^; AGTC Gene Technology Company Ltd) as the paired control. In the normal control group (CON), the right eyes were sham injected without infusing any drug as “treatment” and the left eyes were left intact.

### Histological evaluation of GFP expression

The eyes with rAAV_2_-CMV-GFP injection were enucleated at 1, 3, and 6 months and then fixed in 4% paraformaldehyde for 1 h at 4 °C. After washing thoroughly, the eyes were cut through the pars plana and the posterior sections with retinae were embedded in optimal cutting temperature tissue fluid (Maixin Bio,Led, FuZhou,China) for sectioning. Serials of 10-μm frozen sections were observed with a fluorescence microscope (Olympus, BX51, Japan).

### Reverse transcriptase-PCR assessment

Normal and diabetic rat eyes were enucleated at 1, 3, and 6 months, and the retinae were quickly collected and transferred into lysis buffer. Total RNA was isolated using a Triozol kit (Invitrogen Life Technologies, Shanghai, China), according to the manufacturer’s instructions. First-strand cDNA was synthesized from 0.1 μl of total RNA in 20 μl buffer containing 4 μl MgCl_2_, 1 μl oligo(dT) or random hexamer, 2 μl deoxy-ribonucleoside triphosphate (dNTP), 1 μl AMV reverse transcriptase, and 0.5 μl RNase inhibitor. Based on the manufacturer’s instructions, the reaction mixture was incubated at 30 °C for 10 min, 42 °C for 1 h, heat inactivated at 99 °C for 5 min, 5 °C for 5 min, and then the cDNAs pooled from two reactions with different primers. PCR was performed with 4 μl of pooled cDNA in 20 μl buffer containing 0.5 μl DNA polymerase, and 4 μl of the primers. The primer sequences for *VEGF*, *hPEDF*, and glyceraldehyde phosphate dehydrogenase (*GAPDH*; synthesis by TAKARA Biotechnology CO, LTD, Dalian, China) are as follows; *VEGF*: forward primer 5′-GCA CCC ACG ACA GAA GG-3′, reverse primer 5′-TGA ACG CTC CAG GAT TTA-3′, fragment length 416 bp; *PEDF*: forward primer 5′-CAG AAG AAC CTC AAG AGT GCC-3′, reverse primer 5′-CTT CAT CCA AGT AGA AAT CC-3′, fragment length 310 bp; *GAPDH*: forward primer 5′-ACC ACA GTC CAT GCC ATC AC-3′, reverse primer 5′-TCC ACC ACC CTG TTG CTG TA-3′, fragment length 452 bp. The reaction was cycled as follows: 2 min at 94 °C; 30 cycles of 30 s at 94 °C, 30 s at 57 °C and 1 min at 72 °C, and a final extension of 10 min at 72 °C. PCR products were run on a 1.5% agarose gel.

### Western blot analysis

Normal and diabetic rat eyes were enucleated at 1, 3, and 6 months and the retinae dissected free from surrounding tissue and then homogenized by sonication. The insoluble pellet was removed by centrifugation at 10,000× g for 15 min, and the protein concentration of the supernatant measured using a Coomassie brilliant blue protein assay (Jiancheng Bioengineering Institute, Nanjing, China). The soluble proteins (100 µg) were then resolved by sodium dodecyl sulfate PAGE (SDS–PAGE; 12% polyacrylamide gel for hPEDF, intercellular adhesion molecule-1 (ICAM-1) and occludin, 15% polyacrylamide gel for VEGF) and electrotransferred to a Hybond ECL nitrocellulose membrane (Amersham International, Piscataway, NJ). The membrane was blotted with polyclonal anti-hPEDF antibody (Boster Biotechnology LTD, Wuhan, China), polyclonal anti-VEGF antibody (Boster Biotechnology), anti-ICAM-1 antibody (1:500; Boster Biotechnology), and anti-occludin antibodies (1:1,000; Zymed Laboratory, San Francisco, CA). The concentrations of primary antibodies were 1:100. The membranes were stripped and re-immunostained with an antibody specific to β-actin (1:5,000 dilution; Sigma) as a normalization procedure. The densities of bands were measured with FluorChem version 2.0 imaging system (Alpha Innotech Corporation, San Leandro, CA) and normalized by β-actin as semiquantitation of protein.

### Measurement of blood–retinal barrier breakdown

BRB breakdown was quantified by measuring albumin leakage from retinal blood vessels using the Evans blue albumin method, as described previously [14] with minor modifications. Briefly, Evans blue dye (Sigma) was dissolved in normal saline (30 mg ml^−1^). The rats were anesthetized with ketamine (80 mg kg^−1^) by intraperitoneal injection and the dye (45 mg kg^−1^) injected over 10 s through the caudal vein. The rats were kept on a warm pad for 2 h to ensure complete dye circulation. Two minutes after the injection of Evans blue, 0.2 ml blood was drawn from tail vein to obtain the initial Evans blue plasma concentration. Subsequently, at 15-min intervals, 0.1 ml blood was drawn up to 2 h after injection to obtain the time-averaged Evans blue plasma concentration. At exactly 2 h after infusion, 0.2 ml blood was drawn to obtain the final Evans blue plasma concentration, after which the chest cavity was opened and the rats transcardially perfused via the left ventricle for 2 min (120 mmHg) with 37 °C 1% paraformaldehyde in citrate buffer (pH 4.2) to fix the retinae and clear excess dye from the blood vessels. Immediately after perfusion, the eyes were enucleated and the retinae dissected free from surrounding tissue. After thoroughly drying the retinae, they were weighed, and the Evans blue dye was extracted from the tissue by incubating each sample in 120 μl formamide (Sigma, ShangHai, China) for 18 h at 70 °C. The extract was centrifuged at 81,500× g for 45 min at 4 °C, and then the absorbance in 50 μl of the supernatant was measured at 620 nm and 740 nm. The concentration of Evans blue was calculated from a standard curve and normalized according to the retinal dry weight and expressed in ml plasma×g retinal dry wt^−1^ h^−1^.

### Statistical analysis

All values were given as the mean±standard deviation. One-way ANOVA and Pearson correlation analysis were used for statistical analyses. A p value of ≤0.05 was considered statistically significant. All data were analyzed using SPSS 13.0 (SPSS Inc., Chicago, IL).

## Results

### Expression of GFP in retina mediated by rAAV_2_ vector

**Figure 1:**
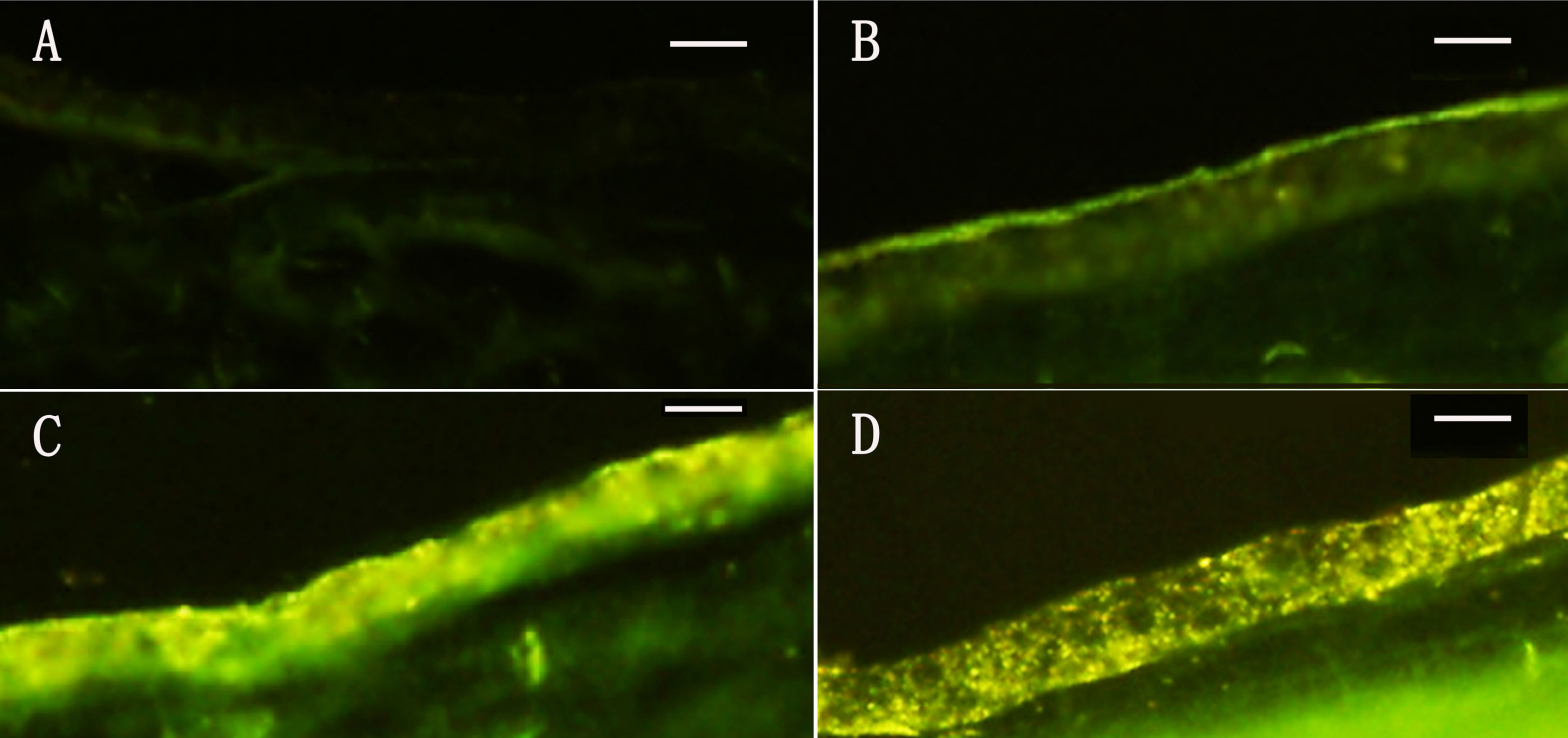
Expression of green fluorescent protein in retinae mediated by recombinant adeno-associated virus vector. **A**: No fluorescence signals were found in normal retinae. **B**: One month later, fluorescence signals were detected in the ganglion cell layer of retinae injected with recombinant adeno-associated virus vector-cytomegalovirus-green fluorescent protein. **C**, **D:** The area of fluorescence extended to most of the retinae after 3 months (**C**) and after 6 months (**D**), fluorescence signals had been detected in all retinae. The orientation of the sections was from superior to inferior. The bar is 200 μm, and magnification is 200×.

GFP has the property of autoradiation of green fluorescence. Using a fluorescence microscope, GFP expression was evaluated after 1, 3 and 6 months of rAAV_2_-CMV-GFP injection. As shown in Figure 1, no hyperfluorescence signal was observed in normal retinae. After 1 month, hyperfluorescence signals were detected in the ganglion cell layer of retinae with rAAV_2_-CMV-GFP injection. Gradually, the area of fluorescence extended to most retinae after 3 months. Finally, hyperfluorescence signals were detected in all retinae after 6 months.

### *PEDF* expression in the retina of diabetic rats after vector injection

**Figure 2:**
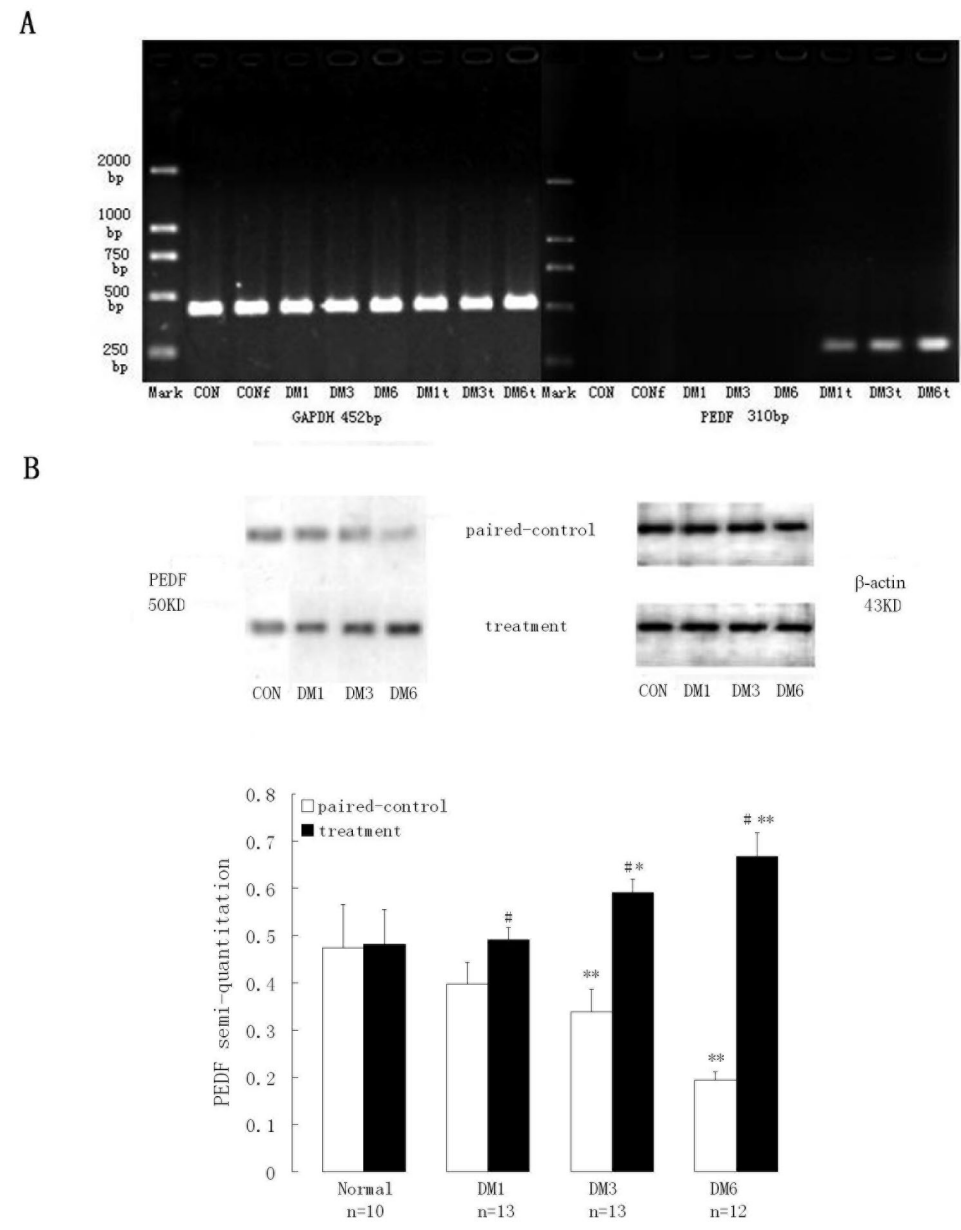
Expression of pigment epithelium-derived factor in retinae after intravitreal injection. **A**: The expression of human pigment epithelium-derived factor (*hPEDF)* mRNA in injected retinae was enhanced and increased over time. No expression were detected in the uninjected control rats. **B**: The protein expression of PEDF decreased in the paired control retinae but significantly increased in treated eyes (p<0.01) compared to paired controls or normal control eyes (p<0.01). Values of treatment were compared to that of normal control *p value <0.05, ** p value<0.01. Values of treatment were compared to that of paired control, # p value <0.01. Abbreviations: DM1 represents diabetes 1 month, DM3 represents diabetes 3 months, DM6 represents diabetes 6 months, CON represents normal control group, CONf represents sham-injected normal control group, t represents treatment.

Expression of *hPEDF* mRNA of diabetic rats and normal control rats was determined by RT–PCR at 1, 3, and 6 months after the injection of rAAV_2_-CMV-hPEDF. Bands about 310 bp corresponding to the *hPEDF* mRNA primer were not detected in normal eyes and paired control eyes, while *hPEDF* mRNA primer expression was identified in the retinae of rAAV_2_-CMV-hPEDF injected eyes and significantly increased over time (p<0.05 n=13, 13, 12, respectively; Figure 2A), reaching a maximum at the 6 months.

Western blot analysis also showed that the expression of PEDF increased significantly in vector-injected eyes compared to the paired control eyes (p<0.01), which showed a decreased PEDF, and normal control eyes (p<0.01). These results were consistent with findings on the expression of PEDF at the mRNA level (Figure 2B, Table 1).

**Table 1:** Expression of hPEDF mRNA

**Groups**	**Paired control**	**Treatment**
CON	0	0
DM1	0	0.34±0.03
DM3	0	0.47±0.04*
DM6	0	0.62±0.05*

The asterisk indicated compared to 1 month p value<0.05. Abbreviations: CON represents normal control group, DM1 represents diabetes 1 month, DM3 represents diabetes 3 months, DM6 represents diabetes 6 months.

### rAAV_2_-CMV-hPEDF injections downregulated expression of *VEGF*

**Figure 3:**
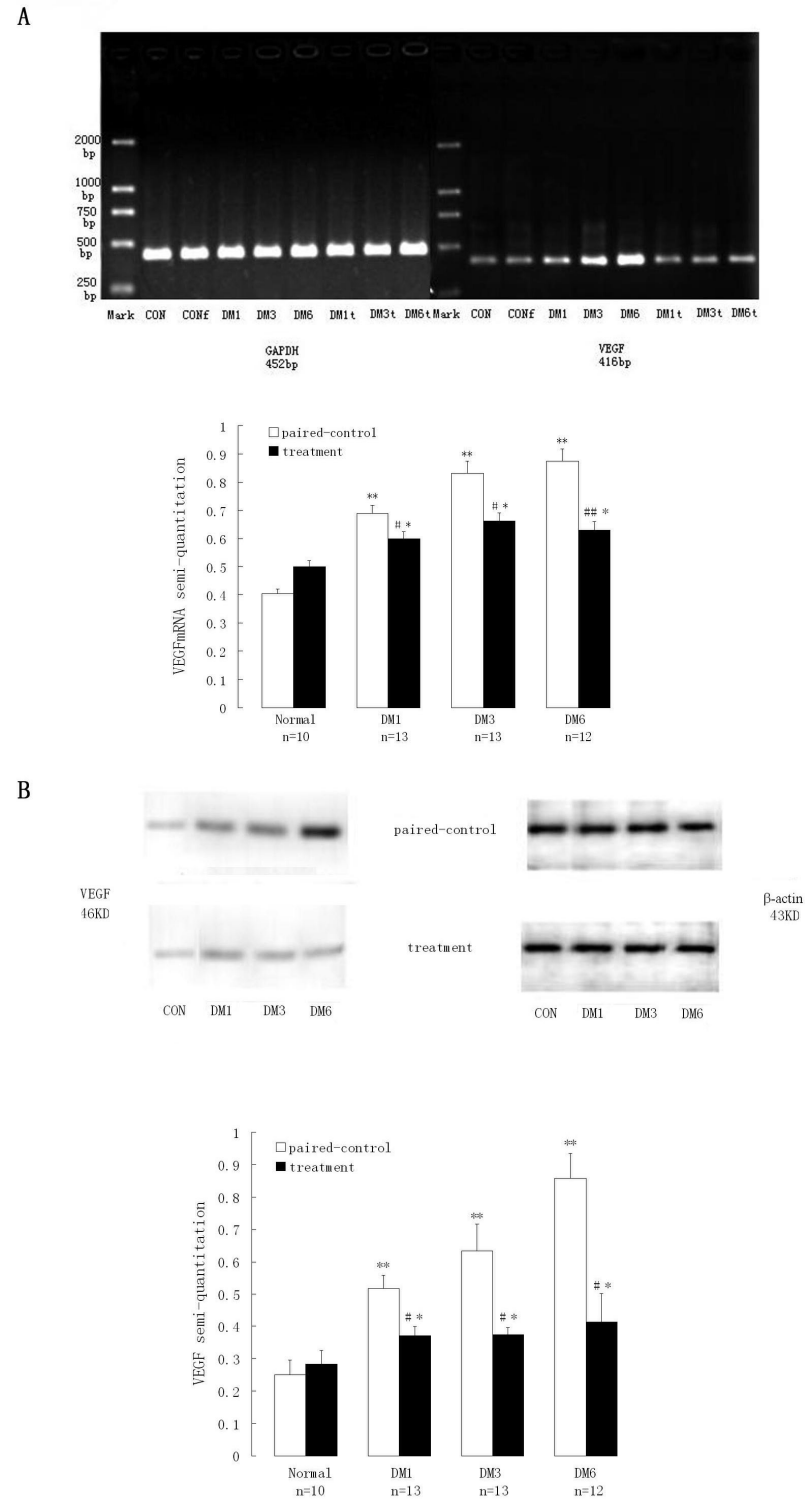
Expression of vascular endothelial growth factor in retinae after intravitreous injection. **A**: Vascular endothelial growth factor (*VEGF*) mRNA in paired control retinae increased with time to almost twice that in the normal control eyes after 6 months. Similarly, the amount of *VEGF* mRNA in the retinae of all treatment groups was statistically higher than the amount in normal retinae but was significantly lower than the amount in the respective paired control eyes (p<0.01). **B**: The expression of VEGF protein in treated retinae paralleled the expression of *VEGF* mRNA. Values of treatment were compared to that of normal control * p value <0.05, ** p value<0.01. Values of treatment were compared to that of paired control # p value <0.01. Abbreviations: DM1 represents diabetes 1 month, DM3 represents diabetes 3 months, DM6 represents diabetes 6 months, CON represents normal control group, CONf represents sham-injected normal control group, t represents treatment.

To assess the relationship between *VEGF* and *PEDF*, we analyzed the expression of *VEGF* in retinae, at both mRNA and protein levels, at 1, 3, and 6 months after vector injection, by using RT–PCR and western blots. *VEGF* mRNA in paired control retinae increased with time to almost twice that in the normal control eyes after 6 months. Similarly, the amount of *VEGF* mRNA in the retinae of all treatment groups was statistically higher than that in the normal retinae (p<0.05, Figure 3A) but was significantly lower than that in the respective paired control eyes (p<0.01). Furthermore, a significant negative correlation between the expression of *VEGF* mRNA and *PEDF* mRNA (r=-0.732, p<0.01) was observed.

The expression of VEGF protein in rAAV_2_-CMV-hPEDF-injected retinae showed similar trends to that of *VEGF* mRNA (Figure 3B), which indicated that overexpression of *hPEDF* in the retina downregulated the expression of *VEGF* at both mRNA and protein levels. In the treated retinae, the value of VEGF against PEDF was nearly equal to that in the normal control and was lower relative to the paired control retinae. However, the ratio of VEGF:PEDF in paired control retinae increased with time to almost eight times that of the normal control’s value after 6 months (Table 2).

**Table 2:** Expression of VEGF in the retinae

**Groups**	**VEGF protein**		**VEGFmRNA**		**VEGFp/PEDFp**	
**Paired control**	**Treatment**	**Paired control**	**Treatment**	**Paired control**	**Treatment**
CON	0.25±0.04	0.28±0.04	0.40±0.02	0.50±0.02	0.53	0.58
DM1	0.52±0.04**	0.37±0.03#*	0.69±0.03**	0.60±0.02#	1.3	0.76
DM3	0.63±0.08**	0.38±0.02#*	0.83±0.04**	0.66±0.03#	1.85	0.64
DM6	0.86±0.08**	0.41±0.09#**	0.87±0.04**	0.63±0.03##	4.42	0.61

Values of treatment were compared to that of normal control * p value <0.05, ** p value<0.01. Values of treatment were compared to that of paired control # p value <0.05, ## p value<0.01. PC=paired control. Abbreviations: T represents treatment, CON represents normal control group, DM1 represents diabetes 1 month, DM3 represents diabetes 3 months, DM6 represents diabetes 6 months, VEGF represents vascular endothelial growth factor, PEDF represents pigment epithelium-derived factor, VEGFp represents VEGFprotein, PEDFp represents PEDF protein 1.

### hPEDF upregulated retinal occludin but downregulated retinal *ICAM-1*

**Figure 4:**
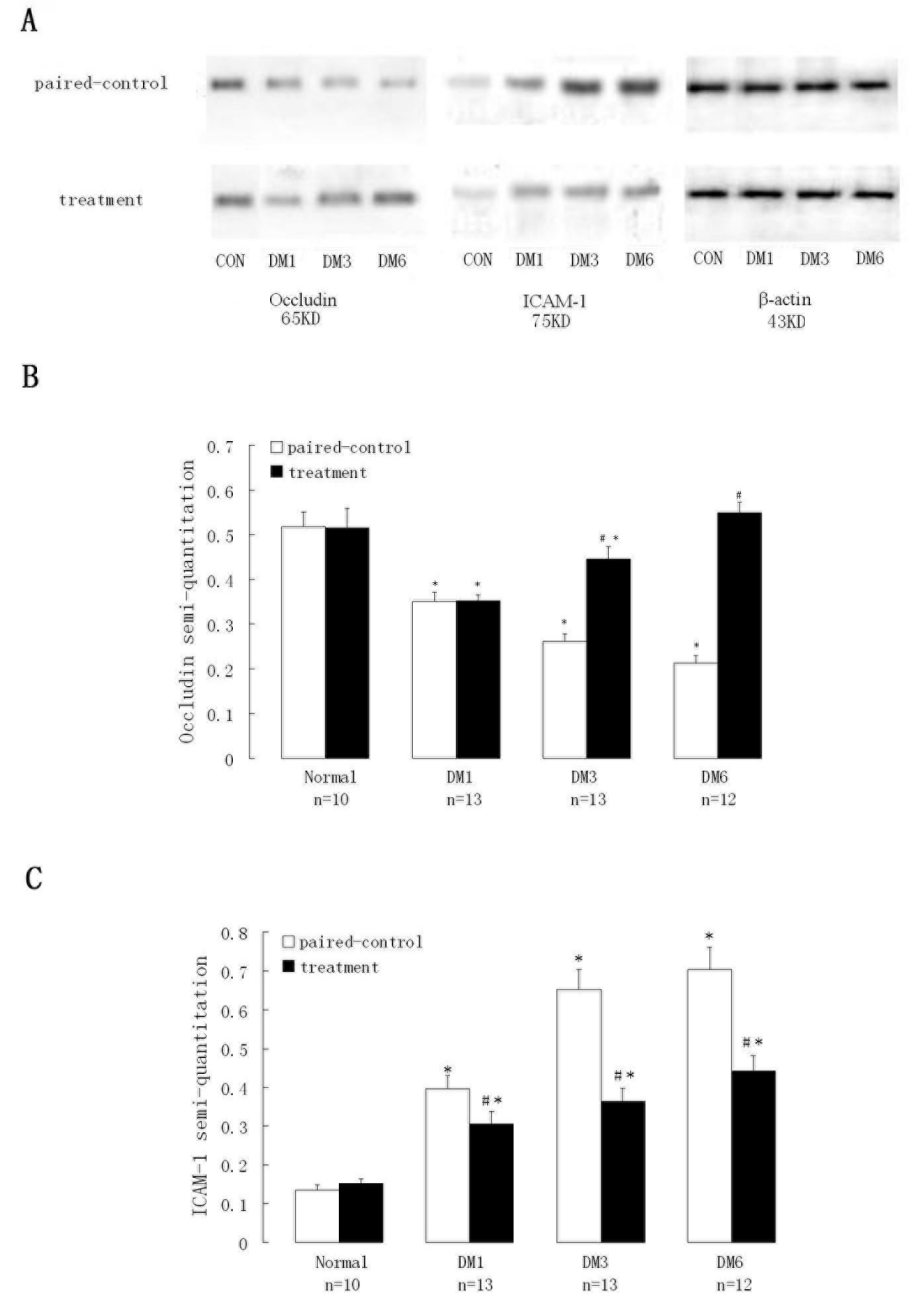
The protein levels of occludin and intercellular adhesion molecule-1 in paired control retinae significantly decreased and increased, respectively, over time. **A**: Western blots had been used to assess occludin and intercellular adhesion molecule-1 (ICAM-1) protein levels in retinae. **B**: The expression of occludin in treated eyes increased significantly at months 3 and 6 compared with paired control eyes. **C**: The expression of ICAM-1 in treated eyes was obviously lower than that in the paired control eyes (p<0.01) but still higher than that in normal control eyes (p<0.01). *Values of treatment were compared to that of normal control, p value <0.05. #Values of treatment were compared to that of paired control, p value <0.01. Abbreviations: DM1 represents diabetes 1 month, DM3 represents diabetes 3 months, DM6 represents diabetes 6 months, CON represents normal control group, CONf represents sham-injected normal control group, t represents treatment.

Western blot was used to assess the effect of hPEDF on occludin and ICAM-1 protein levels after retinal hPEDF gene transfection. Although retinal occludin in treated eyes did not differ significantly from the levels of paired control eyes after 1 month, at 3 and 6 months its level in rAAV_2_-CMV-hPEDF-injected eyes increased significantly compared with paired control eyes (Figure 4) and reached levels similar to that seen in the normal eyes at 6 months. In contrast, the retinal occludin level in paired control eyes was significantly lower than that in the treatment and normal eyes. On the other hand, the expression of ICAM-1 increased in paired controls and treated retinae compared with its expression in normal eyes (p<0.05). Its level remained significantly lower in the injected retinae (p<0.01) compared with paired controls (Table 3) .There was a significant negative correlation between ICAM-1 and occludin (r=-0.754, p<0.01).

**Table 3:** Expression of ICAM-1 and Occludin in the retinae

**Groups**	**ICAM-1**		**Occludin**	
**Paired control**	**Treatment**	**Paired control**	**Treatment**
CON	0.14±0.01	0.15±0.01	0.52±0.03	0.52±0.04
DM1	0.40±0.02*	0.31±0.01#*	0.35±0.02*	0.35±0.01*
DM3	0.65±0.02*	0.36±0.01#*	0.26±0.02*	0.45±0.03*#
DM6	0.70±0.02*	0.44±0.03#*	0.21±0.02*	0.55±0.02#

*Values of treatment were compared to that of normal control, p value <0.05. #Values of treatment were compared to that of paired control, p value <0.01. Abbreviations: PC represents paired control, T represents treatment, CON represents normal control group, DM1 represents diabetes 1 month, DM3 represents diabetes 3 months, DM6 represents diabetes 6 months, ICAM-1 represents intercellular adhesion molecule-1.

### rAAV_2_-CMV-hPEDF injections suppressed blood–retinal barrier breakdown

**Figure 5:**
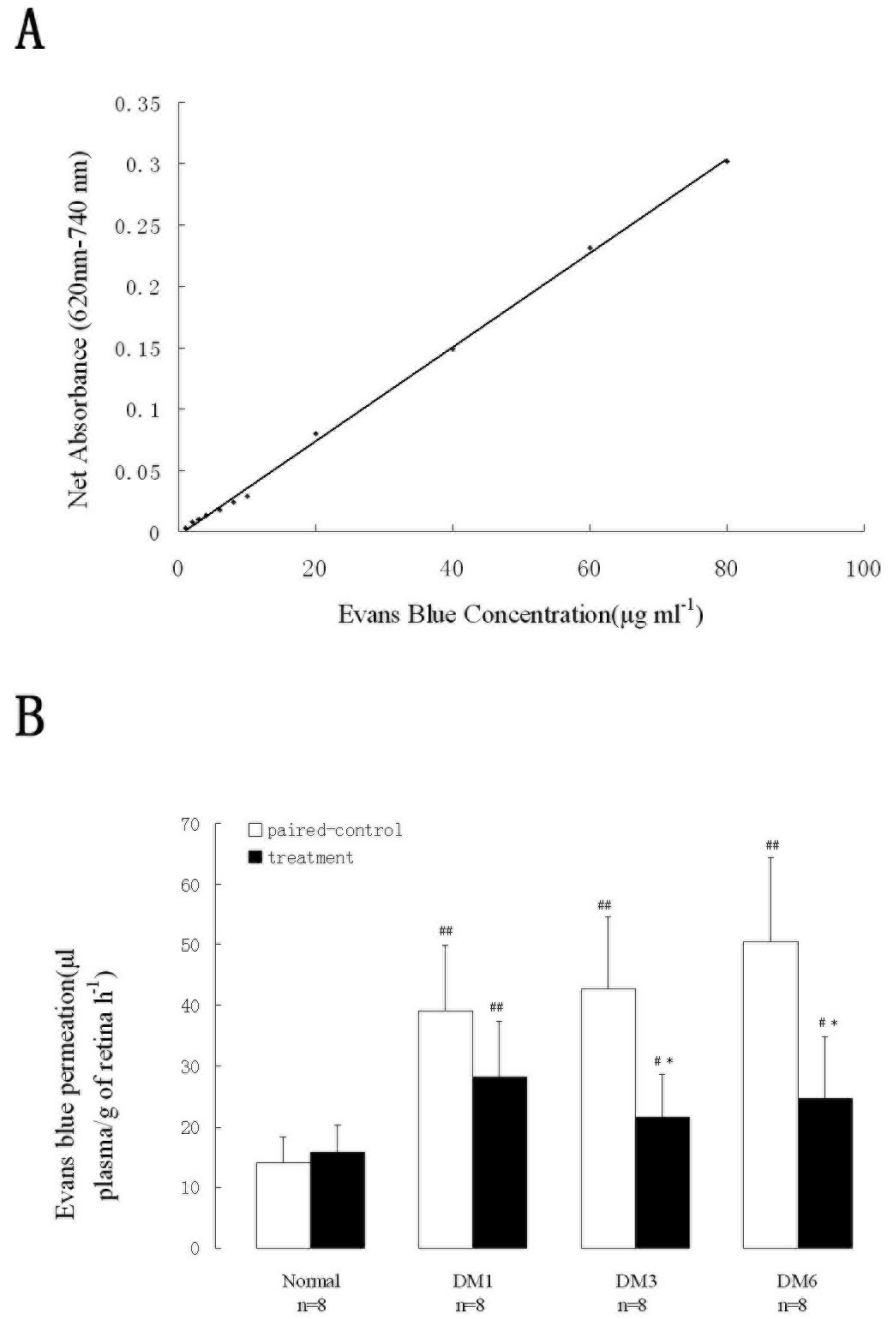
Measurement of blood–retinal barrier breakdown by Evans blue. **A**: Bar graph show standard curve for Evans blue concentration in formamide (background-subtracted absorbance at 620–740 nm; concentration from 1 to 80 μg/ml). **B**: Histogram shows retinal Evans blue leakage in diabetic rats at different times. *Values of treatment were compared to that of normal control * p value <0.05, ** p value<0.01. Values of treatment were compared to that of paired control # p value <0.05, ## p value<0.01

To determine the permeability of the retinal vasculature in diabetic rats and the effect of PEDF on the BRB, we intravenously injected rats with Evans blue albumin on the last day of months 1, 3, and 6. Our standard curves shown in Figure 5A confirm the reliable linear relationship between background-subtracted absorbance (620–740 nm) and Evans blue concentration in formamide from 1 to 80 μg ml^−1^ (r*=*0.999, regression equation y=0.0038×–0.003).

Leakage from the retinal vasculature became more and more severe with the progression of diabetes in the paired control eyes (Figure 5B) and it reached a maximum by the month 6 (50.48±13.79) with a fourfold increase compared with that in normal control eyes (14.04±4.54, p<0.01). The damage to the BRB was less severe in the treatment group compared with the paired control group, especially at the 3 and 6 months (p<0.05). Nevertheless, Evans blue levels in the treatment group were all significantly higher than levels seen in normal control retinae (p<0.05).

## Discussion

Our study demonstrated that single intravitreal injection of rAAV-PEDF relieved BRB breakdown in STZ-induced diabetic rats for 6 months. The effect was associated with the downregulation of retinal *VEGF* mRNA and ICAM-1 expression and concomitant upregulation of occludin expression. rAAV_2_-CMV-hPEDF partly ameliorated the retinal VEGF/PEDF imbalance and thus reduced the vascular permeability and subsequently restored BRB.

Diabetic macular edema is a common pathological feature in DR and responsible for vision loss. BRB breakdown, a characteristic sign of early DR, and the subsequent increase in vascular permeability are believed to play major roles in the development of DME and progression of DR. To date, the exact mechanisms underlying the pathogenesis of the changes of BRB in DR are unclear.

Accumulating clinical evidence has suggested that VEGF plays an important role in DME in early diabetes [10,15,16]. VEGF is a potent vascular permeability factor [17], which significantly increased endothelial permeability (approximately twofold) after 24 h co-incubation with retinal capillary endothelial cells [18], and caused BRB breakdown after sustained intravitreal release in rabbits [19,20]. Previous studies have shown that the levels of VEGF and VEGF receptor were higher in DR [21,22]. In the early stages of STZ-induced diabetes in rats, retinal *VEGF* mRNA levels dramatically increased and were correlated with retinal vascular permeability. However, this early BRB breakdown was successfully prevented by VEGF TrapA40, a soluble VEGF receptor Flt/Fc [23]. In this study, we have confirmed the association between the increases in retinal VEGF levels and BRB breakdown in animals with induced diabetes, suggesting that the overexpression of retinal VEGF induces BRB breakdown.

PEDF is an endogenous angiogenic inhibitor in the eye and can counterbalance the angiogenic effect of VEGF and suppress neovascularization in PDR and age-related macular degeneration (AMD) [24–27]. Clinical studies have shown that decreased expression of PEDF in the retina and vitreous was associated with DME [10]. High levels of PEDF might reduce vascular leakage induced by VEGF [11] or diabetes [18], suggesting that PEDF is involved in the regulation of vascular permeability. Therefore PEDF therapy may be effective not only for PDR but also for DME.

We consider an ideal theraputic strategy for DME treatment would be to develop an approach involving single administration of vectors to sustain the long-term expression of a suitable therapeutic gene. rAAV vectors are highly efficient gene delivery systems that can facilitate long-term transduction [13,24]. Although AAV-PEDF has been used to inhibit retinal and choroidal neovascularization induced by laser or hypoxia [13,27], to our knowledge, this is the first time for the application of AAV-PEDF in eyes of STZ-induced diabetic animals. Indeed, our study has shown that PEDF expression in the retina lasted for 6 months after rAAV_2_-CMV-hPEDF injection, which potentially enables clinicians to avoid repeated intravitreal injections. In addition, we demonstrated that intravitreal injections of rAAV_2_-CMV-hPEDF suppressed BRB breakdown by downregulating the expression of *VEGF* in diabetic rats. In our study, retinal *VEGF* mRNA/protein expression and BRB breakdown decreased in rAAV_2_-CMV-hPEDF-transfected eyes compared with the contralateral control eyes injected with rAAV_2_-CMV-GFP. The retinal balance of VEGF/PEDF was partially corrected in these diabetic rats by downregulation of *VEGF* mRNA expression in the retina, which is consistent with previous reports on the reduction of vascular leakage via the blockade of VEGF with PEDF [11,18]. The exact mechanism(s) by which PEDF reduces vascular leakage through inhibition of VEGF production is still under investigation.

VEGF-mediated upregulation of *ICAM-1*, an adhesion receptor for leukocytes, may contribute to the observed retinal vascular changes, such as vascular leakage and local nonperfusion in early diabetic retinopathy [28,29]. This conclusion is based on two lines of evidence. First, previous studies have demonstrated that the expression of both retinal *VEGF* mRNA [23] and ICAM-1 [30] were upregulated in rodent models of diabetic retinopathy and ICAM-1 levels were decreased by suppressing VEGF activity associated with a decrease in BRB breakdown and leukostasis [31]. Second, studies in nondiabetic rats found that retinal *ICAM-1* was upregulated in response to VEGF [32] and the overexpression of *ICAM-1* mRNA induced by VEGF via the phospholipase c/nuclear factor-kB (PLC/ NF-kB) pathway in endothelial cells [33]. Inhibition of ICAM-1 activity significantly suppressed VEGF-induced hyperpermeability and leukostasis. These data suggest that ICAM-1 is a crucial mediator of the VEGF effect on vascular permeability [34]. In our study, retinal ICAM-1 decreased in the eyes receiving rAAV_2_-CMV-hPEDF injection, suggesting that PEDF decreased *ICAM-1* expression in the diabetic retina.

The tight junctions between retinal vascular endothelial cells constitute an essential structural component of the BRB. We observed that the levels of occludin, a barrier protein, decreased by around 50% to 60% between 3 and 6 months after induction of diabetes, which is consistent with the results of Antonetti and colleagues [35]. VEGF not only decreases occludin expression [18] but also stimulates its phosphorylation and redistribution [36,37] by activating the phosphatidylinositol 3-kinase/protein kinase C (PKC) isoform pathway [38], which subsequently induces elevated vascular permeability. This implies that a lowering and redistribution of retinal occludin induced by overexpression of VEGF may be responsible for BRB breakdown in early DR [35]. Ho and coworkers [39] reported that PEDF prevented the redistribution of occludin in retinal pigment epithelium (RPE) cells from being damaged by oxidants. Our study showed a significant negative relationship between retinal VEGF and occludin. Intravitreal injection of rAAV_2_-CMV-hPEDF significantly increased retinal occludin expression in STZ-induced diabetic rats, with a concomitant reduction in retinal vascular permeability. We suggest, therefore, that hPEDF may reduce vascular leakage partly by preserving retinal occludin content in these rats through inhibition of VEGF. These results suggest that transgenic expression of rAAV_2_-CMV-hPEDF may be a useful strategy for long-term preventive or adjunctive therapy for DME.

Although the findings reported here have important clinical implications for DR therapy, the safety issues and immune response induced by vectors should be studied further. Recently, Li and colleagues found that intravitreal injection of AAV vectors in mice generated a humoral immune response against AAV capsid, which suppressed vector expression upon re-administration via the same route into the partner eye [40]. Application of an AAV vector with intravitreal injection would be limited if a similar immune response is found in humans. However, this conclusion is worthy of further investigation because the vitreous cavity and subretinal space are immune-privileged sites [41], BRB isolates them from the main circulation, and findings in animals cannot be simply extrapolated because humans possess a more complicated immune system. Many factors could affect the immune response against AAV-mediated ocular gene transfer in diabetic eyes, such as vitreous hemorrhages and neovascularization, which compromise the immune privilege of the eye. Further studies need to be performed to address these issues.
